# Higher Prevalence of Metformin-Induced Vitamin B_12_ Deficiency in Sulfonylurea Combination Compared with Insulin Combination in Patients with Type 2 Diabetes: A Cross-Sectional Study

**DOI:** 10.1371/journal.pone.0109878

**Published:** 2014-10-09

**Authors:** Donghoon Kang, Jae-Seung Yun, Sun-Hye Ko, Tae-Seok Lim, Yu-Bae Ahn, Yong-Moon Park, Seung-Hyun Ko

**Affiliations:** 1 Division of Endocrinology and Metabolism, Department of Internal Medicine, St. Vincent's Hospital, College of Medicine, The Catholic University of Korea, Seoul, Korea; 2 Department of Epidemiology and Biostatistics, Arnold School of Public Health, University of South Carolina, Columbia, South Carolina, United States of America; The University of Hong Kong, Hong Kong

## Abstract

Long-term and high-dose treatment with metformin is known to be associated with vitamin B_12_ deficiency in patients with type 2 diabetes. We investigated whether the prevalence of B_12_ deficiency was different in patients treated with different combination of hypoglycemic agents with metformin during the same time period. A total of 394 patients with type 2 diabetes treated with metformin and sulfonylurea (S+M group, n = 299) or metformin and insulin (I+M group, n = 95) were consecutively recruited. The vitamin B_12_ and folate levels were quantified using the chemiluminescent enzyme immunoassay. Vitamin B_12_ deficiency was defined as vitamin B_12_≤300 pg/mL without folate deficiency (folate>4 ng/mL). The mean age of and duration of diabetes in the subjects were 59.4±10.5 years and 12.2±6.7 years, respectively. The mean vitamin B_12_ level of the total population was 638.0±279.6 pg/mL. The mean serum B_12_ levels were significantly lower in the S+M group compared with the I+M group (600.0±266.5 vs. 757.7±287.6 pg/mL, *P*<0.001). The prevalence of vitamin B_12_ deficiency in the metformin-treated patients was significantly higher in the S+M group compared with the I+M group (17.4% vs. 4.2%, *P* = 0.001). After adjustment for various factors, such as age, sex, diabetic duration, duration or daily dose of metformin, diabetic complications, and presence of anemia, sulfonylurea use was a significant independent risk factor for B_12_ deficiency (OR = 4.74, 95% CI 1.41–15.99, *P* = 0.012). In conclusion, our study demonstrated that patients with type 2 diabetes who were treated with metformin combined with sulfonylurea require clinical attention for vitamin B_12_ deficiency and regular monitoring of their vitamin B_12_ levels.

## Introduction

Metformin, which belongs to the biguanide class, is one of the most generally used oral hypoglycemic agents. It has been used for more than 50 years and was approved by the US Food and Drug Administration (FDA) in 1994 [1]. Currently, many clinical practice guidelines for patients with type 2 diabetes, including the American Diabetes Association (ADA), the European Association for the Study of Diabetes (EASD), and the Korean Diabetes Association (KDA), recommend that metformin treatment should begin at the time of diagnosis of diabetes with lifestyle modification in the absence of contraindications [2]–[4]. Moreover, metformin is preferentially selected for combination therapy with sulfonylurea or insulin to achieve the glycemic target goal. Thus, the use of metformin has steadily increased worldwide. According to data using the IMS Health National Disease and Therapeutic Index of the U.S., more than 50% of treatment visits before 2012 were associated with metformin [5].

Long-term metformin treatment is a known pharmacological cause of vitamin B_12_ deficiency, as was evident within the first 10–12 years [6]–[10] after it started to be used, and is associated with decreased serum folate concentrations [11], [12]. In addition, metformin treatment may be an iatrogenic cause for the exacerbation of peripheral neuropathy in patients with type 2 diabetes who exhibit depressed vitamin B_12_ levels and elevated fasting total homocysteine and methylmalonic acid (MMA) levels [11]. This is clinically important because patients with diabetes often suffer from neurological symptoms, such as numbness, paresthesia, and impaired vibration sensation and proprioception. These clinical symptoms can easily be considered symptoms of diabetic peripheral neuropathy, and anticonvulsants or tricyclic antidepressants (TCAs) are frequently used as drugs to control these uncomfortable symptoms. However, the symptoms of vitamin B_12_ deficiency can be easily misunderstood as diabetic peripheral neuropathy, which can lead to unnecessary drug use and misdiagnosis as other demyelinating nerve diseases.

We previously reported a high prevalence of vitamin B_12_ deficiency in patients with type 2 diabetes treated with metformin, particularly in subjects with a longer duration and higher daily dose of metformin use [13]. However, most patients with diabetes are not treated with metformin monotherapy for glycemic control, and there have been few studies on the effect of combinations of hypoglycemic agents with metformin on vitamin B_12_ deficiency. Thus, we focused on the effect of combinations of metformin with either sulfonylurea or insulin, which are frequently used hypoglycemic agents, on vitamin B12 deficiency during the same time period.

Thus, the aim of this study was to investigate the effect of sulfonylurea or insulin on vitamin B_12_ deficiency when used as a combinatorial therapy with metformin for patients with type 2 diabetes.

## Methods

### Ethics Statement

This study was performed according to the Declaration of Helsinki and approved by the Institutional Review Board of the Catholic University Medical Centre. Written informed consent was obtained from all of the participants.

### Study Design and Subjects

Between January 2012 and April 2014, patients with type 2 diabetes, aged 25 to 80 years, who had taken metformin plus sulfonylurea (S+M group) or metformin plus insulin (I+M group) for at least six months were recruited consecutively at the university-affiliated diabetes center of St. Vincent's Hospital in South Korea. To exclude any effects of different treatment periods of sulfonylurea or insulin on the vitamin B_12_ level, we included only those patients who used either insulin plus metformin or sulfonylurea plus metformin during the same time period. A maximum difference of three months of treatment for each combination was allowed for inclusion in this study.

Patients with newly diagnosed type 2 diabetes, type 1 diabetes, pernicious anemia, or decreased renal function (serum creatinine level>1.7 mg/dL for males or >1.5 mg/dL for females) and pregnant patients were excluded. Patients with a previous history of vitamin B_12_ injections, gastrectomy, colectomy, inflammatory bowel disease, or chronic heavy alcohol drinker and vegetarians were also excluded. Patients with severe medical illnesses, such as severe infection, sepsis, malignancies, liver cirrhosis, heart failure or renal failure, were also excluded from this study.

The medication history of the enrolled subjects was evaluated using a dietary supplement questionnaire, and the medication history included over-the-counter multivitamins, calcium supplements, histamine-2 receptor blockers (H2 blockers) and proton pump inhibitors (PPIs). To analyze the effects of sulfonylurea dosage on vitamin B_12_ deficiency, we classified the patients into 4 groups according to the dosages of their sulfonylurea regimens as follows: **Group 1**: glimepiride 1 mg, gliclazide 80 mg, gliclazide MR 30 mg, and glibenclamide 5 mg (n = 40); **Group 2**: glimepiride 2 mg, gliclazide 160 mg, gliclazide MR 60 mg, and glibenclamide 10 mg (n = 116); **Group 3**: glimepiride 3 mg, gliclazide 240 mg, gliclazide MR 90 mg, and glibenclamide 15 mg (n = 56); and **Group 4**: glimepiride 4 mg, gliclazide 320 mg, gliclazide MR 120 mg, and glibenclamide 20 mg (n = 87)[14], [15].

The patients were defined “alcohol” drinkers if their average consumption was 1 to 2 drinks per day (>1 g alcohol/day after converting the average frequency and amount of alcoholic beverages consumed into units of pure alcohol (in grams) consumed per day[16]. The patients were also divided into current smokers, ex-smokers or non-smokers according to their smoking habits. Hypertension was defined as systolic blood pressure ≥140 mmHg, diastolic blood pressure ≥90 mmHg, or any use of antihypertensive medications [17].

### Assessments and Outcome Measures

The primary outcome was biochemical vitamin B_12_ deficiency, which was determined by the serum vitamin B_12_ concentrations. The serum vitamin B_12_ and folate levels were quantified using a chemiluminescent enzyme immunoassay (Immulite 2000; Siemens, Berlin, Germany). We defined biochemical vitamin B_12_ deficiency as serum levels ≤300 pg/mL without folate deficiency [18], [19]. In the absence of recent anorexia or fasting, a serum folate concentration <2 ng/mL was diagnostic of folate deficiency. Anemia was defined as Hb <13 g/dL for males and <12 g/dL for females based on the WHO guidelines [20]. The blood glucose level was measured using an automated enzymatic method, and the HbA_1_c level was determined through high-performance liquid chromatography (HPLC-723 G8, Tosoh, Tokyo, Japan). The total cholesterol, triglyceride, and HDL-cholesterol levels were measured enzymatically using an automatic analyzer (Hitachi 736-40, Hitachi, Tokyo, Japan). The measurement of microalbuminuria was performed using immunoturbidimetry (Hitachi 7600-110) through random spot urine collection, and the albumin-to-creatinine ratio (ACR) was calculated. Diabetic nephropathy was defined as ACR≥30 µg/mg creatinine [2]. Diabetic retinopathy was assessed from retinal photographs at baseline, and the findings were reviewed by a board-certified ophthalmologist and classified by the absence or presence of diabetic retinopathy. Diabetic neuropathic symptoms were defined by the presence of typical symptoms, such as pain, burning or aching, prickling sensations, hypoesthesia or numbness in both of the lower legs or feet, through a questionnaire [21]–[23].

### Statistical analyses

We used SAS version 9.2 (SAS Institute, Inc., Cary, NC, USA) for statistical analyses. The clinical characteristics and parameters are expressed as the means ± standard deviation (SD) or numbers (percentages). Pearson's Chi-square tests were used to test the differences in the proportion of categorical variables, and independent *t*-tests were used to evaluate the differences between the means of two continuous variables. One-way ANOVAs were used to evaluate the differences in the means of the continuous variables between the sulfonylurea groups. Pearson correlation analyses were performed to examine the linear relationship between the serum vitamin B_12_ levels and the duration of metformin use. The variables that were found to be statistically significant in the univariable analysis or reported to affect the vitamin B_12_ levels were included in the multivariable analysis. A multivariable logistic regression analysis was performed to assess the independent association of each factor with vitamin B_12_ deficiency. *P* values <0.05 were considered to be statistically significant.

## Results

After exclusion during the study period, 394 eligible patients were enrolled and completed the evaluation. The clinical characteristics of the patients are shown in Table 1. Of the 394 patients with type 2 diabetes, the mean age and duration of diabetes were 59.4±10.5 years and 12.2±6.7 years, respectively. In this study, 45.4% of the patients were males, 51.0% had hypertension, and the mean HbA_1_c level was 7.8±1.5% at baseline. The mean vitamin B_12_ level was 638.0±279.6 pg/mL, and 56 patients (14.2%) showed vitamin B_12_ deficiency (Table 1).

**Table 1:** Baseline clinical characteristics of the subjects.

Characteristic	Total subjects (n = 394)
Age (years)	59.4±10.5
Men, n (%)	179 (45.4)
Diabetic duration (years)	12.2±6.7
Alcohol (yes, %)	94 (23.9)
Smoking (yes, %)	69 (17.5)
BMI (kg/m^2^)	24.8±3.2
Hypertension (yes, %)	201 (51.0)
Diabetic retinopathy (yes, %)	109 (27.7)
Diabetic neuropathic symptom (yes, %)	86 (21.8)
Diabetic nephropathy (yes, %)	132 (33.5)
Duration of metformin use (months)	82.6±49.4
Daily dose of metformin (mg)	1305.5±449.4
Hypertensive medication (yes, %)	242 (61.4)
Use of statin (yes, %)	212 (53.8)
Over-the-counter multivitamin(yes, %)	46 (11.7)
Calcium supplement (yes, %)	18 (4.6)
H2 blocker or PPI (yes, %)	30 (7.6)
FBS (mg/dL)	145.5±53.9
Creatinine (mg/dL)	0.8±0.2
TC (mg/dL)	166.9±37.4
TG (mg/dL)	131.5±115.9
HDL-cholesterol (mg/dL)	49.0±23.3
LDL-cholesterol (mg/dL)	95.9±40.7
HbA_1_c (%)	7.8±1.5
Vitamin B_12_ deficiency, n (%)	56 (14.2)
Vitamin B_12_ (pg/mL)	638.0±279.6
Serum folate (ng/mL)	9.9±5.7
Anemia^*^(yes, %)	61 (15.5)
Hemoglobin (g/dL)	13.9±5.3
MCV (fL)	89.0±5.0
MCH (fL)	30.7±1.8
MCHC (fL)	34.2±1.3

The data are shown as the means (SD) or n (%). ^*^Hb<13 g/dL for men and <12 g/dL for women (WHO guidelines); BMI, body mass index; H2 blocker, histamine 2 receptor blocker; PPI, proton pump inhibitor; FBS, fasting blood sugar; TC, total cholesterol; TG, triglyceride; MCV, mean corpuscular volume; fL, femtoliter.

Among the total population, 299 patients were treated with metformin combined with sulfonylurea (S+M group), and 95 patients were treated with metformin and insulin (I+M group). Compared with the S+M group, the subjects in the I+M group showed a longer diabetic duration, higher BMI, higher prevalence of diabetic retinopathy and nephropathy, and higher HbA_1_c level (Table 2). Remarkably, the mean vitamin B_12_ level was significantly lower in the S+M group compared with the I+M group (600.0±266.5 vs. 757.7±287.6 pg/mL, *P*<0.001). In addition, the prevalence of vitamin B_12_ deficiency was significantly higher in the S+M group than in the I+M group (17.4 vs. 4.2%, *P*<0.005). However, the duration of metformin, the daily dose of metformin, and the prevalence of diabetic neuropathic symptoms and anemia were not significantly different between the two groups (Table 2).

**Table 2:** Characteristics of the subjects according to combination treatment with metformin.

Characteristic	S+M (n = 299)	I+M (n = 95)	*P* value
Age (years)	59.7±10.3	58.2±10.8	.223
Men, (%)	143 (47.8)	36 (37.9)	.091
Diabetic duration (years)	11.2±6.3	15.5±7.2	<.001
Alcohol (yes, %)	80 (26.8)	14 (14.7)	.017
Smoking (yes, %)	53 (17.7)	16 (16.8)	.741
BMI (kg/m^2^)	24.5±3.2	25.5±3.2	.008
Hypertension (yes, %)	152 (50.8)	49 (51.6)	.900
Diabetic retinopathy (yes, %)	67 (22.4)	42 (44.2)	<.001
Diabetic neuropathic symptom (yes, %)	64 (21.4)	22 (23.2)	.422
Diabetic nephropathy (yes, %)	81 (27.1)	51 (53.7)	<.001
Duration of metformin use (months)	80.5±47.0	89.1±56.1	.180
Daily dose of metformin (mg)	1276.1±451.2	1397.9±432.9	.175
Use of statin (yes, %)	154 (51.5)	58 (61.1)	.104
Over-the-counter multivitamin(yes, %)	34 (11.4)	12 (12.6)	.740
Calcium supplement (yes, %)	14 (4.7)	4 (4.2)	.848
H2 blocker or PPI (yes, %)	20 (6.7)	10 (10.5)	.220
FBS (mg/dL)	144.6±52.3	148.3±58.9	.582
Creatinine (mg/dL)	0.8±0.2	0.8±0.3	.288
TC (mg/dL)	168.1±37.0	163.3±38.3	.275
TG (mg/dL)	132.4±127.6	128.7±67.0	.789
HDL-cholesterol (mg/dL)	50.1±24.3	45.5±19.3	.057
LDL-cholesterol (mg/dL)	97.5±42.0	91.0±36.3	.183
HbA_1_c (%)	7.6±1.3	8.7±1.8	<.001
Vitamin B_12_ deficiency, n (%)	52 (17.4)	4 (4.2)	.001
Vitamin B_12_ (pg/mL)	600.0±266.5	757.7±287.6	<.001
Serum folate (ng/mL)	9.8±5.6	10.3±6.0	.444
Anemia^*^(yes, %)	42 (14.0)	19 (20.0)	.176
Hemoglobin (g/dL)	14.1±6.0	13.4±1.8	.318
MCV (fL)	89.1±5.1	89.0±4.4	.949
MCH (fL)	30.7±1.7	30.6±1.8	.551
MCHC (fL)	34.2±1.3	34.2±1.2	.893

The data are presented as the means (SD) or n (%).^*^Hb<13 g/dL for men and <12 g/dL for women (WHO guidelines); BMI, body mass index; H2 blocker, histamine 2 receptor blocker; PPI, proton pump inhibitor; FBS, fasting blood sugar; TC, total cholesterol; TG, triglyceride; MCV, mean corpuscular volume; fL, femtoliter.

S+M: Metformin + Sulfonylurea; I+M: Metformin + Insulin.

The mean vitamin B_12_ levels of the normal B_12_ level group and the vitamin B_12_ deficiency group were 704.5±244.3 pg/mL and 237.0±46.0 pg/mL, respectively (*P*<0.005). Compared with the subjects without vitamin B_12_ deficiency, the patients with vitamin B_12_ deficiency were older in age, used more sulfonylurea, and had lower levels of HbA_1_c and hemoglobin (Table 3). In addition, the patients with vitamin B_12_ deficiency demonstrated a higher frequency of anemia but not megaloblastic type anemia than the normal vitamin B_12_ level group (26.8% vs. 13.6%, *P* = 0.017).

**Table 3:** Characteristics of the two selected groups according to vitamin B_12_ deficiency.

Characteristic	Vitamin B_12_ deficiency (−)	Vitamin B_12_ deficiency (+)	*P* value
n	338	56	
Age (years)	58.7±10.4	63.3±10.3	.003
Men, (%)	156 (46.2)	23 (41.1)	.563
Diabetic duration (years)	12.2±6.9	12.7±5.5	.548
Alcohol (yes, %)	80 (23.7)	14 (25.0)	.866
Smoking (yes, %)	60 (17.8)	9 (16.1)	.826
BMI (kg/m^2^)	24.8±3.3	24.4±2.9	.330
Hypertension (yes, %)	170 (50.3)	31 (55.4)	.564
Sulfonylurea use (yes, %)	244 (72.2)	52 (92.9)	<.001
Insulin use (yes, %)	91 (26.9)	4 (7.1)	.001
Diabetic retinopathy (yes, %)	97 (28.7)	12 (21.4)	.332
Diabetic neuropathic symptom (yes, %)	70 (20.7)	16 (28.6)	.221
Diabetic Nephropathy (yes, %)	117 (34.6)	15 (26.8)	.287
Duration of metformin use (months)	79.2±49.5	102.6±44.5	.001
Daily dose of metformin (mg)	1252.8±425.9	1623.2±459.9	<.001
Hypertensive medication (yes, %)	205 (60.7)	37 (66.1)	.463
Use of statin (yes, %)	179 (53.0)	33 (58.9)	.470
Over-the-counter multivitamin(yes, %)	38 (11.2)	8 (14.3)	.502
Calcium supplement (yes, %)	16 (4.7)	2 (3.6)	1.000
H2 blocker or PPI (yes, %)	24 (7.1)	6 (10.7)	.410
FBS (mg/dL)	146l5±55l4	139.0±43.2	.248
Creatinine (mg/dL)	0.8±0.2	0.8±0.2	.685
TC (mg/dL)	168.0±37.7	160.5±34.8	.145
TG (mg/dL)	131.2±121.9	133.1±69.6	.869
HDL-cholesterol (mg/dL)	49.6±24.1	45.8±17.4	.160
LDL-cholesterol (mg/dL)	96.6±41.6	92.0±35.1	.379
HbA_1_c (%)	7.9±1.6	7.4±1.4	.006
Vitamin B_12_ (pg/mL)	704.5±244.3	237.0±46.0	<.001
Serum folate (ng/mL)	9.9±5.7	9.7±5.6	.747
Anemia^*^(yes, %)	46 (13.6)	15 (26.8)	.017
Hemoglobin (g/dL)	14.1±5.7	13.1±1.5	.010
MCV (fL)	89.1±5.1	89.0±4.2	.800
MCH (fL)	30.8±1.8	30.4±1.7	.150
MCHC (fL)	34.3±1.2	33.9±1.7	.106

The data are presented as the means (SD) or n (%).^*^Hb<13 g/dL for men and <12 g/dL for women (WHO guidelines); BMI, body mass index; H2 blocker, histamine 2 receptor blocker; PPI, proton pump inhibitor; FBS, fasting blood sugar; TC, total cholesterol; TG, triglyceride; MCV, mean corpuscular volume; fL, femtoliter.

Of the total subjects involved in the present study, 61 (15.5%) had anemia, and 14 (4.7%) of the patients also had vitamin B_12_ deficiency. Of the patients with B_12_ deficiency, 15 (26.8%) patients were anemic, but 46 (13.6%) of the patients without B_12_ deficiency were anemic (*P* = 0.017, Table 3). Only four of the total subjects (1.02%) had a mean corpuscular volume (MCV)>100 fL, and only two patients had anemia (Hb 12.4 g/dL in males, Hb 10.4 g/dL in females).

The duration of treatment and daily dose of metformin were significantly higher in the patients with vitamin B_12_ deficiency compared with the patients without vitamin B_12_ deficiency (Table 3). However, the presence of diabetic complications and the duration of diabetes were not different between the two groups.

**Figure 1:**
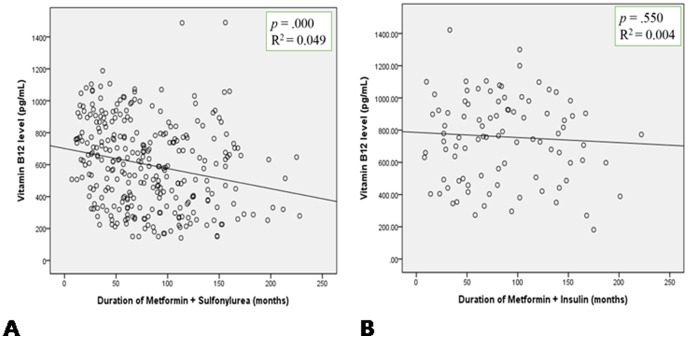
Correlation between the vitamin B_12_ levels and the duration of metformin treatment in two different groups. A: S+M group, B: I+M group. R^2^: coefficient of determination.

The correlation between the serum vitamin B_12_ level and the duration of metformin use was evaluated. The vitamin B_12_ levels showed a significant negative correlation with the duration of metformin treatment in the S+M group (R^2^ = 0.049, *P*<0.001, Fig. 1A) but not in the I+M group (R^2^ = 0.004, *P* = 0.550, Fig. 1B).

After adjusting for confounding factors, such as age, sex, diabetic duration, duration and daily dose of metformin treatment, the presence of hypertension or anemia, the HbA_1_c level, and diabetic complications, the multivariable logistic regression analysis revealed that sulfonylurea use was significantly associated with vitamin B_12_ deficiency in patients with type 2 diabetes (OR = 4.74, 95% CI 1.41–15.99, *P* = 0.012, Table 4).

**Table 4:** Logistic regression analysis for potential risk factors of Vitamin B_12_ deficiency among patients with type 2 diabetes.

Risk factor	OR (95% CI)	*P* value
Male gender	0.63 (0.27–1.48)	.284
Age (per year)	1.05 (1.01–1.09)	.028
Sulfonylurea use	4.74 (1.41–15.99)	.012
Diabetic duration (per year)	0.93 (0.86–1.00)	.056
Duration of metformin use (year)		.009
<5 years	1	
5 to 9 years	4.72 (1.82–12.27)	.001
≥10 years	6.74 (1.94–23.50)	.003
Daily dose of metformin (mg)		<.001
<1000 mg/day	1	
1000 to 1999 mg/day	3.64 (0.43–30.66)	.234
≥2000 mg	31.15 (3.44–282.20)	.002
Use of H2 blocker or Proton pump inhibitor	1.23 (0.39–3.89)	.731
Over-the-counter multivitamin use	1.39 (0.51–3.77)	.516
Calcium use	0.38 (0.07–2.22)	.282
HbA_1_c (%)	0.74 (0.56–0.99)	.044
Anemia	2.03 (0.87–4.71)	.101

**Figure 2:**
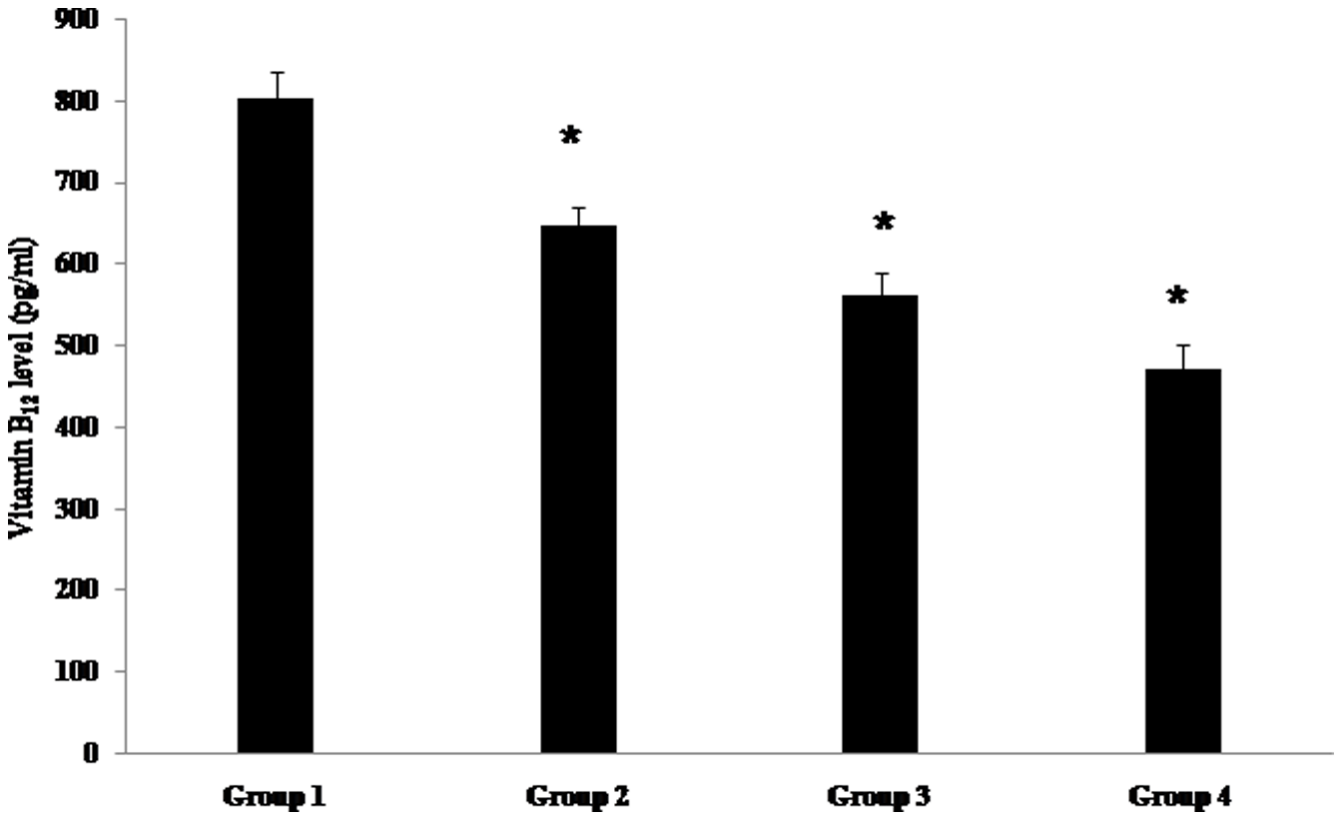
The mean value of vitamin B_12_ according to daily sulfonylurea dosage (* *P*<0.05 vs. Group 1). **Group 1**: glimepiride 1 mg, gliclazide 80 mg, gliclazide MR 30 mg, and glibenclamide 5 mg (n = 40); **Group 2**: glimepiride 2 mg, gliclazide 160 mg, gliclazide MR 60 mg, and glibenclamide 10 mg (n = 116); **Group 3**: glimepiride 3 mg, gliclazide 240 mg, gliclazide MR 90 mg, and glibenclamide 15 mg (n = 56); and **Group 4**: glimepiride 4 mg, gliclazide 320 mg, gliclazide MR 120 mg, and glibenclamide 20 mg (n = 87).

To investigate the effects of the dosage of sulfonylurea on vitamin B_12_ deficiency, we performed further analyses according to the mean daily dosages of sulfonylurea. There were no significant differences between the clinical variables of the patients in each of the four groups at the beginning of the study with the exception of diabetic duration and HbA_1_c level (Table 5). When we classified the patients into four groups according to daily dosages of sulfonylurea, we found that the vitamin B_12_ level was significantly lower in the higher dosage group (Group 4) than in Group 1, *P*<0.001; Fig. 2, Table 5). The prevalence of vitamin B_12_ deficiency also increased with increasing sulfonylurea dosage. Compared to Group 1, the percentage of vitamin B_12_ deficiency was approximately 15-fold higher in the patients of Group 4 (2.5% vs. 39.1%, *P*<0.001, Table 5). Even after adjusting for age, sex, diabetic duration, hypertension, alcohol drinker, duration and daily dose of metformin, hemoglobin level, HbA_1_c level, and presence of anemia in the multivariable logistic regression, this analysis revealed that the highest dosage of sulfonylurea was significantly associated with the presence of vitamin B_12_ deficiency in patients with type 2 diabetes (OR 20.3, 95% CI 2.39–171.9, *P* = 0.006, Table 6).

**Table 5:** Clinical characteristics according to daily dosage of sulfonylurea.

	Group 1 (n = 40)	Group 2 (n = 116)	Group 3 (n = 56)	Group 4 (n = 87)	*P* value
Age	59.8±10.5	58.9±10.2	60.6±11.9	60.0±9.8	0.768
Men, (%)	21 (52.5)	57 (49.1)	25 (44.6)	40 (46.0)	0.855
Diabetic duration (years)	9.23±5.2	10.6±6.0	12.9±6.0	11.8±6.0	0.017
Alcohol (yes, %)	8 (20.0)	40 (34.5)	10 (17.9)	20 (23.0)	0.064
BMI (kg/m^2^)	24.1±3.1	24.7±3.4	24.5±2.9	24.5±3.1	0.729
Hypertension (yes, %)	26 (65.0)	52 (44.8)	31 (55.4)	37 (42.5)	0.093
Duration of metformin use (months)	71.1±45.9	73.4±47.0	92.2±45.7	86.7±46.8	0.026
Daily dose of metformin (mg)	906.3±342.9	1181.0±395.3	1369.6±394.0	1512.6±453.7	<0.001
FBS (mg/dL)	134.1±25.0	138.8±40.8	148.5±57.3	154.9±68.0	0.081
Creatinine (mg/dL)	0.79±0.2	0.80±0.2	0.78±0.2	0.83±0.2	0.651
HbA_1_c (%)	7.03±0.6	7.47±1.2	7.61±1.2	7.90±1.7	0.006
Vitamin B_12_ (pg/mL)	801.8±214.2	647.3±238.9	561.0±216.9	471.3±281.4	<0.001
Vitamin B_12_ deficiency (yes, %)	1 (2.5)	9 (7.8)	8 (14.3)	34 (39.1)	<0.001
Serum folate (ng/mL)	11.8±6.3	9.36±5.2	9.78±5.8	9.40±5.5	0.120
Anemia (yes, %)*	3 (7.5)	12 (10.3)	12 (21.4)	15 (17.2)	0.055
Hemoglobin (g/dL)	16.7±1.61	13.9±1.4	13.5±1.6	13.5±1.6	0.032

The data are presented as the means (SD) or n (%).^*^Hb<13 g/dL for men and <12 g/dL for women (WHO guidelines); BMI, body mass index; FBS, fasting blood sugar.

**Group 1**; glimepiride 1 mg, gliclazide 80 mg, gliclazide MR 30 mg, glibenclamide 5 mg, **Group 2**; glimepiride 2 mg, gliclazide 160 mg, gliclazide MR 60 mg, glibenclamide 10 mg, **Group 3**; glimepiride 3 mg, gliclazide 240 mg, gliclazide MR 90 mg, glibenclamide 15 mg, **Group 4**; glimepiride 4 mg, gliclazide 320 mg, gliclazide MR 120 mg, glibenclamide 20 mg.

**Table 6:** Relationship between daily dosage of sulfonylurea regimen and vitamin B_12_ deficiency.

	Odds ratio (95% CI)			
	Model 1	Model 2	Model 3	Model 4
Group 1	1.0	1.0	1.0	1.0
Group 2	3.46 (0.42–28.5)	4.15 (0.49–34.7)	3.43 (0.40–29.3)	2.68 (0.31–23.3)
Group 3	6.13 (0.73–51.8)	7.57 (0.88–65.4)	4.67 (0.52–42.2)	3.96 (0.43–36.6)
Group 4	27.6 (3.57–213.0)	39.5 (4.9–317.0)	23.4 (2.79–195.0)	20.3 (2.39–171.9)
*P* value	<0.001	<0.001	<0.001	<0.001

Multivariable logistic regression analysis models were adjusted as follows: model 1: sex, age; model 2: model 1 + diabetes duration, hypertension, HbA_1c_; model 3: model 2 + duration of metformin, daily dose of metformin; model 4: model 3 + alcohol, hemoglobin, presence of anemia.

**Group 1**; glimepiride 1 mg, gliclazide 80 mg, gliclazide MR 30 mg, glibenclamide 5 mg, **Group 2**; glimepiride 2 mg, gliclazide 160 mg, gliclazide MR 60 mg, glibenclamide 10 mg, **Group 3**; glimepiride 3 mg, gliclazide 240 mg, gliclazide MR 90 mg, glibenclamide 15 mg, **Group 4**; glimepiride 4 mg, gliclazide 320 mg, gliclazide MR 120 mg, glibenclamide 20 mg.

## Discussion

In this study, we found a more significant association between the combined treatment of sulfonylurea plus metformin and a higher prevalence of vitamin B_12_ deficiency in patients with type 2 diabetes compared with that found for the insulin plus metformin combination therapy. We also demonstrated that vitamin B_12_ deficiency was significantly correlated with the duration of metformin use. After adjusting for confounding factors, the sulfonylurea combination was significantly associated with metformin-related vitamin B_12_ deficiency compared with the insulin combination during the same time period. This study provides the first analysis of metformin-associated vitamin B_12_ deficiency in response to combined hypoglycemic medications.

Metformin treatment with lifestyle modifications is generally recommended as the first-line treatment for type 2 diabetes [2]–[4]. If the glycemic control status does not reach the target range, additional hypoglycemic medications should be added to the metformin therapy based on the patients' clinical characteristics. With a steady increase in the number of patients with diabetes, a longer life expectancy of diabetic patients, and the clinical benefits of intensive glycemic control, oral hypoglycemic agents or insulin are consequently expected to be used more often in combination with metformin.

Vitamin B_12_ deficiency is one of the clinically important side effects of metformin treatment. Approximately 6–10% and up to 30% of patients receiving metformin for diabetes treatment have been reported to experience reduced vitamin B_12_ absorption [23]–[27], and the serum vitamin B_12_ levels were decreased in 14% to 30% of metformin-treated patients [11], [28], [29]. A recent study from the National Health and Nutrition Examination Survey (NHANES) revealed that 5.8% of patients with diabetes using metformin presented with vitamin B_12_ deficiency (serum vitamin B_12_ level ≤200 pg/dL) compared with 2.4% of patients with diabetes not using metformin and 3.3% of patients without diabetes [25].

Patients with metformin-induced vitamin B_12_ deficiency exhibit some neurological symptoms, such as paresthesias, impaired vibration sensation and proprioception, which are a potential result of neurological damage. These symptoms may be mistaken as diabetic peripheral neuropathy [7].A previous study reported an association between low vitamin B_12_ levels and poor nerve conduction velocities with poorer responses to light touch via monofilament detection [30]. This may lead patients to use unnecessary anticonvulsants or tricyclic antidepressants [7], [31], [32]. Because vitamin B_12_-associated neuropathy is a reversible and treatable condition, the early detection and treatment of vitamin B_12_ deficiency is clinically important in patients with diabetes using metformin [32], [33].

Based on our results, 14.2% of the subjects showed vitamin B_12_ deficiency. The patients treated with metformin plus sulfonylurea (S+M group) showed a significantly higher prevalence of vitamin B_12_ deficiency compared with those treated with metformin plus insulin (I+M group) for the same time period. However, the prevalence of diabetic neuropathic symptoms or anemia was not different between the two groups. In addition, the patients with vitamin B_12_ deficiency were older in age and used metformin for a longer duration and at a higher daily dose; in addition, more patients with vitamin B_12_ deficiency used sulfonylurea, and approximately a two-fold higher number of patients with vitamin B_12_ deficiency had anemia compared with the patients without vitamin B_12_ deficiency. The diabetic duration and the presence of diabetic microvascular complications did not affect vitamin B_12_ deficiency.

Classically, vitamin B_12_ deficiency is related to megaloblastic anemia (MCV>100 fL) [6], [18]. However, the observed mean MCV level in our subjects with vitamin B_12_ deficiency was not greater than 100 fL, and the prevalence of megaloblastic anemia was approximately 0.5% (n = 2). No difference was found in the mean MCV between the groups with and without vitamin B_12_ deficiency. Thus, the anemia was most likely caused from chronic illness. Previous reports have indicated that up to 30% of vitamin B_12_-responsive disorders have normal MCVs [33]–[37]. Moreover, the masking of the macrocytic expression of megaloblastic anemia by coexisting thalassaemia, iron deficiency and chronic illness has been widely reported [37], [38]. Thus, investigating the red cell distribution width and the reticulocyte index and the careful examination of the blood using a peripheral blood smear may be helpful for distinguishing vitamin B_12_ deficiency-related anemia from anemia due to other causes [37], [38].

In our study population, 86 participants (21.8%) had typical diabetic neuropathic symptoms. The mean diabetic duration and the duration of metformin treatment were 12.2 years and 82.6 months, respectively. Remarkably, the presence of diabetic neuropathic symptoms was not different between the groups with and without vitamin B_12_ deficiency. Although the duration of use and the daily dose of metformin were correlated with vitamin B_12_ deficiency, a long-standing diabetic duration with long-term metformin use may contribute to this lack of difference in the presence of neuropathic symptoms. In addition, diabetic neuropathy, as suggested by typical symptoms, did not reflect the severity of the diabetic neuropathy despite the presence of vitamin B_12_ deficiency.

There is no known mechanism of metformin-induced vitamin B_12_ deficiency. Some hypotheses include bacterial overgrowth in the small intestine, which has been attributed to diabetes mellitus, changes in small bowel motility, changes in bacterial flora, competitive inhibition or inactivation of vitamin B_12_ absorption, or an effect of calcium on cell membranes [39]–[41]. Moreover, none of the studies explain the effect of sulfonylurea on vitamin B_12_ metabolism in metformin-induced vitamin B_12_ deficiency. In the present study, we clearly demonstrated that the mean blood vitamin B_12_ level significantly decreased and that the prevalence of vitamin B_12_ deficiency significantly increased with the daily dosage of sulfonylurea in patients with type 2 diabetes. Remarkably, this finding remained significantly after adjustments for the daily dosage and duration of metformin treatment among the patients taking the maximal sulfonylurea dosage. Therefore, we suggest that sulfonylurea, in contrast to insulin, might affect intestinal vitamin B_12_ absorption or metabolism when combined with metformin particularly in patients taking the maximal dosage for extended periods.

There are some limitations to this study. First, this hospital-based cross-sectional study included a small number of patients with relatively long-standing diabetes. In addition, the assessment of typical diabetic neuropathic symptoms may make it difficult to observe the effect of vitamin B_12_deficiency on diabetic neuropathy. Second, we did not measure the MMA, homocysteine or iron levels. These laboratory examinations could not be performed routinely on an outpatient basis. Finally, this cohort study consisted entirely of an Asian population. Thus, additional studies are required to apply this finding to other ethnic groups.

In conclusion, our data suggested the need for regular vitamin B_12_ monitoring in patients with type 2 diabetes, particularly patients receiving higher daily dosage of sulfonylurea plus metformin treatment for a long time period, even in the absence of hematological abnormalities. Further evaluation is needed to clarify the pathological mechanisms and clinical recommendations for B_12_ supplementation for patients with prolonged metformin therapy in the future.
